# Immunoprotective Efficacy of Six *In vivo*-Induced Antigens against *Actinobacillus pleuropneumoniae* as Potential Vaccine Candidates in Murine Model

**DOI:** 10.3389/fmicb.2016.01623

**Published:** 2016-10-21

**Authors:** Fei Zhang, Sanjie Cao, Zhuang Zhu, Yusheng Yang, Xintian Wen, Yung-Fu Chang, Xiaobo Huang, Rui Wu, Yiping Wen, Qigui Yan, Yong Huang, Xiaoping Ma, Qin Zhao

**Affiliations:** ^1^Research Center of Swine Disease, College of Veterinary Medicine, Sichuan Agricultural UniversityChengdu, China; ^2^Sichuan Science-observation Experiment of Veterinary Drugs and Veterinary Biological Technology, Ministry of AgricultureChengdu, China; ^3^Department of Population Medicine and Diagnostic Sciences, College of Veterinary Medicine, Cornell University, IthacaNY, USA

**Keywords:** *Actinobacillus pleuropneumoniae*, IVI antigens, putative vaccine candidates, humoral immune response, cellular immune response

## Abstract

Six *in vivo*-induced (IVI) antigens—RnhB, GalU, GalT, Apl_1061, Apl_1166, and HflX were selected for a vaccine trial in a mouse model. The results showed that the IgG levels in each immune group was significantly higher than that of the negative control (*P* < 0.001). Except rRnhB group, proliferation of splenocytes was observed in all immunized groups and a relatively higher proliferation activity was observed in rGalU and rGalT groups (*P* < 0.05). In the rGalT vaccinated group, the proportion of CD4+ T cells in spleen was significant higher than that of negative control (*P* < 0.05). Moreover, proportions of CD4+ T cells in other vaccinated groups were all up-regulated to varying degrees. Up-regulation of both Th1 (IFN-γ, IL-2) and Th2 (IL-4) cytokines were detected. A survival rate of 87.5, 62.5, and 62.5% were obtained among rGalT, rAPL_1166, and rHflX group, respectively while the remaining three groups was only 25%. Histopathological analyses of lungs indicated that surviving animals from the vaccinated groups showed relatively normal pulmonary structure alveoli. These findings confirm that IVI antigens used as vaccine candidates provide partial protection against *Actinobacillus pleuropneumoniae* infection in a mouse model, which could be used as potential vaccine candidates in piglets.

## Introduction

*Actinobacillus pleuropneumoniae* (APP) was first isolated from pig lung in 1957 and initially described as *Haemophilus*-like organism, and is one of the most important respiratory pathogens in pigs (Matthews and Pattison, 1961; Fraile et al., 2010). This pathogen causes porcine contagious pleuropneumoniae (PCP), which is a major problem for the pig industry worldwide (Fraile et al., 2010; Jirawattanapong et al., 2010; Fablet et al., 2012; Merialdi et al., 2012; Hillen et al., 2014). To date, 15 different serotypes have been identified and the dominant serotypes are different in the various APP endemic regions of the world (Mittal et al., 1983; Klein et al., 2003; Kaplan and Mulks, 2005; Bosse et al., 2014).

Control of PCP is still a difficult problem worldwide. This disease has long been the subject of numerous studies encompassing many areas like diagnosis, pathogenesis, vaccination, etc. (Jacobsen et al., 2005; Oldfield et al., 2009; Costa et al., 2011; Wei et al., 2012; Lopez-Bermudez et al., 2014; Tobias et al., 2014). Furthermore, studies have found an increasing resistance against antimicrobial agents used in pig pleuropneumonia therapy (Aarestrup and Jensen, 1999; Archambault et al., 2012; Vanni et al., 2012). Routine use of antibiotics in livestock to boost animal growth has inadvertently selected for pathogen resistance; this is thought to be the primary factor behind the rapid increase of resistant APP strain outbreaks observed on pig farms worldwide. This issue has received an increasing amount of attention in developed countries, as well as developing ones such as China.

Commercial inactivated vaccines against APP are used routinely in the porcine agronomy. Moreover, a number of recombinant antigens of APP were demonstrated to be putative vaccine candidates. Most of the current commercial vaccines against this pathogen are traditional inactivated bacterins, which elicit only partial protection, with decreased mortality but not morbidity rates (Lu et al., 2011). Traditionally, inactivated vaccines are developed using one or more serovars, which is (are) selected from the local prevalent strain(s). Often, there are multiple serovars prevalent in one country or district (Mittal et al., 1983; Angen et al., 2008; Hälli et al., 2014; Marois-Crehan et al., 2014; Gottschalk and Lacouture, 2015). Because of a lack of cross-protection between different serovars, it is difficult to control PCP effectively using inactivated bacterins (Rycroft and Garside, 2000). APP virulence factors such as apx toxins, are selected as vaccine candidates and show partial cross-protection among different serovars, which are conserved in APP strains (Wang et al., 2009; Seo et al., 2013). In addition, other virulent factors like AasP autotransporter protein (Oldfield et al., 2009), pilin (Lu et al., 2011) were also studied for their potential as vaccine candidates. A number of *in vivo*-induced (IVI) antigens are up-regulated after infection *in vivo*, which are essential for pathogenesis and survival in host internal environment (Salim et al., 2005). Recently, advances in biotechnology have allowed for the development of techniques such as IVI antigen technology (IVIAT; Handfield et al., 2000), signature-tagged mutagenesis (STM; Fuller et al., 2000), and selective capture of transcribed sequences (SCOTS; Baltes et al., 2007). These techniques were proven to be effective in the study of virulence factors.

A number of IVI antigens were identified in our previous studies (Zhang et al., 2015). To evaluate the potential of IVI antigens as vaccine candidates, six antigens were randomly selected from a list of previously identified IVI antigens. In our previous study, IVIAT was used to screen *in vivo* up-regulation antigens of APP; a number of antigens were identified which were involved in metabolism, replication, transcription regulation, signal transduction, and several function-unknown proteins (Zhang et al., 2015). Six of these were expressed as His-tagged fusion proteins and screened for their potential as vaccine candidates against App in a murine model.

## Materials and Methods

### Animals and Ethics Statement

Six to eight week female BALB/c mice (18–22 g) were purchased from Chengdu Dossy Experimental Animal Co., Ltd. The animal experiments were conducted in compliance with the animal protocol approved by the Veterinary Medical College at Sichuan Agricultural University. All procedures performed in this study involving animals were approved by the Institutional Animal Care and Use Committee of Sichuan Agricultural University (Approval Number BK2014-047), Sichuan, China and followed the guidelines of the National Institutes of Health.

### Bacterial Strains, Media, and Culture Conditions

Recombinant constructs were made using gene recombination technology. *E. coli* DH5α (TIANGEN, China) was used for cloning of recombinants and *E. coli* BL21 (TIANGEN, China) was used to express the proteins encoded-fragments inserted in the expression vectors pET-28a. *E. coli* strains were cultured in Luria–Bertani (LB) medium with kanamycin (50 μg/mL) as needed. APP strains were cultured in Trypticase Soy Agar (TSA) or Trypicase Soy Broth (TSB) (DIFCO Laboratories, USA) with 10% (v/v) fetal calf serum (FBS; SIJIQING, China) and nicotinamide adenine dinucleotide (NAD; 15 μg/mL) at 37°C. APP L20 (serovar 5b reference strain) was purchased from the China Institute of Veterinary Drug Control (Beijing, China).

### Bioinformatics Analysis

**FIGURE 1:**
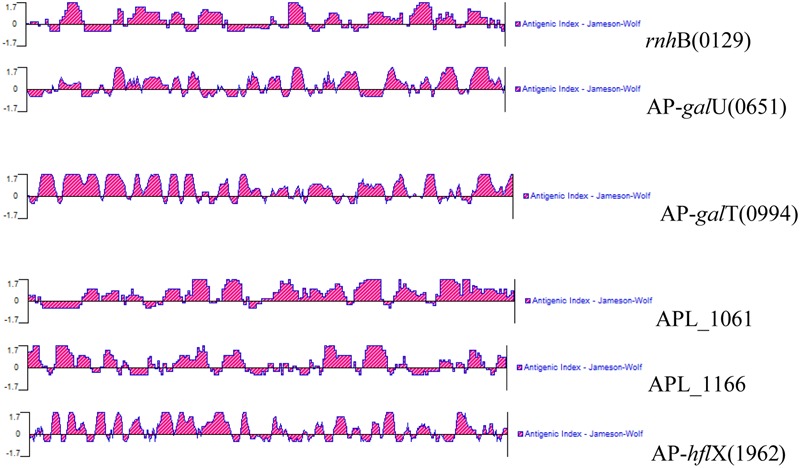
**Antigenic properties of the *in vivo*-induced antigens predicted using a bioinformatics approach**.

Signal peptide cleavage sites of open reading frames (ORFs) were predicted using SignalP 4.1 Server^1^. Before the research was carried out, the antigenicity of these antigens was calculated using bioinformatics prediction software. To determine the antigenicity of the six selected antigens, the complete amino acid sequences of the proteins were predicted using the Protean program in DNAstar; this analysis was performed based on the Jameson–Wolf mathematical model (**Figure 1**). All six selected IVI antigens proved to be highly antigenic, and the results of the prediction are shown in **Figure 1**. The antigenic indices of these proteins were more than 1.7, indicating that these IVI antigens are likely highly antigenic.

### Construction of Expression Recombinant IVI Antigens

Gene sequences encoding IVI antigens were searched using NCBI database. Primers for cloning these sequences were designed using Primer Premier 5 software. The details of the primers, sequences, and products of the IVI genes are listed in **Table 1**. In the primer sequences, the underlined nucleotides mark added restriction sites. APP L20 strain was cultured at 37°C overnight and genome DNA was extracted using genome extraction kit (OMEGA, USA). Using the extracted genome DNA as template, genes of the six IVI antigens were amplified using polymerase chain reaction (PCR). Each amplified gene and vector, pET-28a were both cut by a corresponding restriction enzyme, ligated using T4 DNA ligase (NEB, USA; **Table 1**) and transformed into *E. coli* DH5α. Each clone was confirmed by DNA sequencing.

**Table 1:** Primers used to amplify and clone *A. pleuropneumoniae* IVI gene sequences.

Sequence name	Primer	Primer sequence (5′–3′)	Enzyme	Predicted sizes	Molecular weight (kDa)
galU(0651)	AP1F	5′-CGGGATCCATGAAAGTAATTATTCCGGTAGCGG-3′	*Bam*HI	900	32.48
	AP1R	5′-CCCAAGCTTATAACGTTTTAGCTAATTTTTTA-3′	*Hin*dIII		
AP-*gal*T(0994)	AP2F	5′-CGGGATCCATGAGCCAACAATTTATCCTAAACG-3′	*Bam*HI	1050	40.54
	AP2R	5′-CCCAAGCTTCTATTGATTTTTATAGTGAACGCTG-3′	*Hin*dIII		
AP-*hfl*X(1962)	AP3F	5′-CGGGATCCATGGAATTCCAAACGCTTGCCG-3′	*Bam*HI	760	27.72
	AP3R	5′-ACGTGTAACAGTAAGCTTGCTTCGG-3′	*Hin*dIII		
APL_1166	AP4F	5′-CGGGATCCTTGAAAAAAAGTATTTACGATACCC-3′	*Bam*HI	756	29.31
	AP4R	5′-CCCAAGCTTAATTTTTTTTAACTGCCTTAATA-3′	*Hin*dIII		
rnhB(0129)	AP5F	5′-CGGGATCCATGAGTACAAATTTCATTTATCCTA-3′	*Bam*HI	594	21.42
	AP5R	5′-CCCAAGCTTATAAACCTAAGATTTTCTTCACC-3′	*Hin*dIII		
APL_1061	AP6F	5′-CGGGATCCACGACCGCACAAGCGCAAAATAACG-3′	*Bam*HI	597	22.19
	AP6R	5′-ATAAGAATGCGGCCGCTATTTCAATCTCGTGATGTTTTTC-3′	*Not*I		

### Expression and Purification of the IVI Recombinant Protein

**FIGURE 2:**
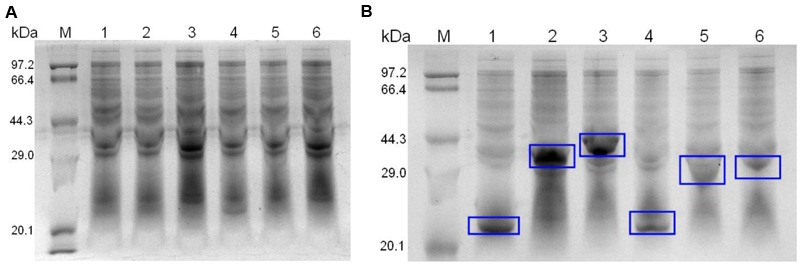
**SDS-PAGE of un-induced **(A)** and induced **(B)** recombinant strains.** The expression proteins are marked with blue rectangles.

For expression, identified recombinant plasmids were extracted from *E. coli* DH5α and transformed into *E. coli* BL21. Clones were cultured in LB and induced with isopropyl-β-D-thiogalactoside (IPTG) for 4 h. Recombinant proteins were purified using Ni-chelating affinity chromatography (BIO-RAD, USA). The purified proteins were examined by 12% SDS-PAGE as previously described (**Figure 2**).

### Western Blotting

Western blotting was performed as described previously (Guo et al., 2015). The membrane was visualized using BeyoECL Star (BEYOTIME, China) and pictures were taken using a ChemiDoc imaging system (BIO-RAD, USA).

### Vaccination and Animal Challenge

To obtain an accurate challenge dose, the LD_50_ was determined using a Reed–Muench method (Reed and Muench, 1938). Animals were divided randomly into seven groups, 15 animals in each group. For the initial vaccination, six groups were immunized subcutaneously with 50 μg rRnhB, rGalU, rGalT, rAPL_1061, rAPL_1166, and rHflX emulsified in the same volume of complete Freund’s adjuvant, respectively. The negative control group was immunized with phosphate buffer solution (PBS) emulsified in complete Freund’s adjuvant. Two weeks following the initial immunization, the mice were given a booster shot of the same dose of antigens and complete Freund’s adjuvant. Before immunization and 2 weeks post-booster, blood samples were obtained by tail bleeding. Two weeks after the booster immunization, all groups of mice were challenged intraperitoneally with a lethal dose of 5 × 10^8^ colony forming unit (CFU) (10 LD50) of App L20. The animals were intensively monitored daily after challenge for the presence and severity of respiratory symptoms and general illness or mortality. All the challenged animals were euthanized; lung tissue was harvested for histopathologic analysis.

### Antibody Determination by Indirect ELISA

IgG levels of serum from each vaccination group were determined by indirect enzyme-linked immunosorbent assay (ELISA) as described previously with a minor modification (Fu et al., 2013). Briefly, the purified proteins were diluted to an identical concentration (w/v; 2.5 μg/mL) in sodium carbonate buffer (PH 9.6). Each well of 96-well microtiter plates (COSTAR, USA) was coated with diluted protein (100 μL) and incubated at 4°C overnight. The plates were incubated with block buffer (BEYOTIME, China) at room temperature (RT) for 1 h then washed three times using washing buffer (300 μL; BEYOTIME, China). Serum samples were diluted (1:100) with primary antibody dilution buffer (BEYOTIME, China), added to the ELISA wells and incubated overnight. The plates were then washed three times. Horseradish peroxidase (HRP)-labeled goat anti-mouse IgG(H+L) was diluted (1:2000) with secondary antibody dilution buffer (BEYOTIME, China) and added (100 μL) onto plates for incubation at RT for 1 h. After washing three times, soluble tetramethylbenzidine (TMB) substrate solution (TIANGEN, China) was added (100 μL), and plates were incubated in the dark at RT for 30 min. The reaction was terminated with 2 M H_2_SO_4_ and absorbance of each well was read at a wavelength of 450 nm in a microplate absorbance reader (BIO-RAD, USA).

### Lymphocyte Proliferation Assay

Lymphocyte proliferation was performed with minor modifications as previously described (Fu et al., 2013). Firstly, spleens were isolated aseptically and processed by gentle disruption using sterile stainless steel screen and the rubber seal of a disposable syringe. Splenocytes were suspended in Roswell Park Memorial Institute (RPMI) incomplete medium (THERMO, USA). Cell suspensions were first processed using Red Blood Cell Lysis Buffer (SOLARBIO, China) according to manufacturer’s instructions. The erythrocyte-free cells were washed three times with Hank’s balanced salt solution (HBSS; THERMO, USA) and then resuspended in complete RPMI medium (THERMO, USA). Cell counting was performed and 100 μL of cells were added (1 × 105 cells) into 96-well culture plates (COSTAR, USA). The cells were stimulated with the recombinant proteins (5 μg/well) or concanavalin A (ConA; SIGMA-ALDRICH, USA) and incubated for 72 h at 37°C in a 5% CO_2_ incubator. Lymphoproliferation assays were performed using 3-(4,5-dimethyl-2-thiazolyl)-2,5-diphenyl-2H-tetrazolium bromide (MTT) Cell Proliferation Assay Kit (BEYOTIME, China), according to the manufacturer’s instructions. MTT solution was added into each well and plates were incubated for another 4 h. Then 100 μL formazan solutions were added to wells and plates were incubated until formazan was dissolved completely. The absorbance was measured at 595 nm using a microplate absorbance reader (BIO-RAD, USA).

### Analysis of the Spleen T-cell Subsets

Two weeks post-booster, aliquots of the splenocytes prepared as mentioned above were subjected to T cells subset analysis by flow cytometry. Briefly, cell counting was performed and the concentration of the spleen cells was adjusted to 1 × 10^6^ cells/mL. Hundred microliters of cell suspension was transferred to another centrifuge tube, and stained with rat anti-mouse CD3-Fluorescein (FITC), R-phycoerythrin/Cyanine 5 (SPRD) (Southern Biotech, USA), rat anti-mouse CD4-R-phycoerythrin (PE) (Southern Biotech), and rat anti-mouse CD8a-PerCP (Southern Biotech) at 4°C for 15 min in the dark. Stained cells were washed in PBS and were resuspended in 450 μL PBS. Resuspended cells were analyzed for T cell subsets by BD fluorescence activated cell sorting (FACS) Calibur flow cytometer (BD, USA) and the results of analysis were collected.

### Detection of Cytokines by ELISA

To compare the levels of Th1 and Th2 cells subsets before vaccination and booster, cytokines (IFN-γ, IL-2, and IL-4) in splenocyte culture supernatant were determined using an ELISA kit (NEOBIOSCIENCE, China) per manufacturer’s instructions.

### Histopathology

Lungs of animals from different groups were isolated and were fixed by immersion in 10% neutral formalin. Immersed tissues were sectioned at 5 μm thickness, and HE-stained for evaluation of histopathology under an Olympus DP71 microscope.

### Immunohistochemical Analysis

Immunohistochemical (IHC) method was conducted to analyze neutrophils and macrophages infiltration in tissues from different groups. IHC staining with monoclonal antibodies (MAbs) was carried out on paraffin wax sections obtained from different mouse lung sections. Two MAbs were used: rabbit anti-mouse CD68 for macrophages and rabbit anti-mouse myeloperoxidase (MPO) for neutrophils (Guge Biotech, Wuhan, China). Sections were treated as described (Oh et al., 2013). The stained IHC sections were examined in 200 × magnification and taken photos. IHC photos were analyzed by Image-Pro Plus 6.0 software. Integrated optical density (IOD) of each photo was determined as positive index.

### Statistical Analyses

All data analyses were performed by SPSS 19.0 software using Student’s *t*-test for the comparison of the differences between different groups. *P*-values of < 0.05 were considered as statistically different and were represented with asterisk. *P*-values of < 0.001 were represented with two asterisks.

## Results

### Expression of Six Recombinant Proteins

**FIGURE 3:**
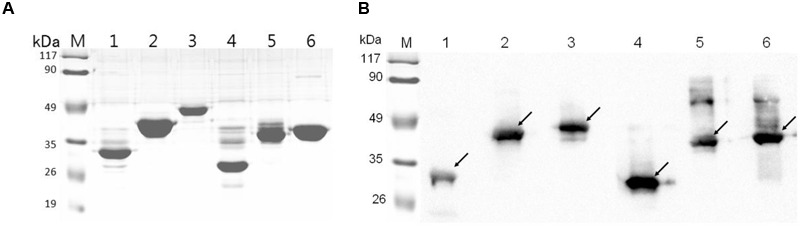
**SDS-PAGE and Western blotting. (A)** SDS-PAGE of six recombinant proteins. **(B)** The results of a western blot of six proteins reacted with corresponding mouse anti-sera. Lane M, protein marker; lanes 1–6, RnhB, GalU, GalT, APL_1061, APL_1166, HflX.

The six recombinant genes were confirmed by sequence analysis (data not shown). The recombinant plasmids were successfully transformed into *E. coli* BL21 and the respective proteins were expressed as His-tagged fusion proteins. SDS-PAGE confirmed that all of these fusion proteins were expressed in *E. coli* BL21 with the expected molecular mass (**Figure 2**). Fusion proteins were purified using Ni-chelating affinity chromatography and determined by SDS-PAGE (**Figure 3A**). Compared with the results of SDS-PAGE for each IVI antigen protein (**Figure 3A**), there was a corresponding exposure stripe on the results of western blotting (**Figure 3B**). This demonstrated that these proteins would react with corresponding anti-sera.

### Humoral Immune Responses

**FIGURE 4:**
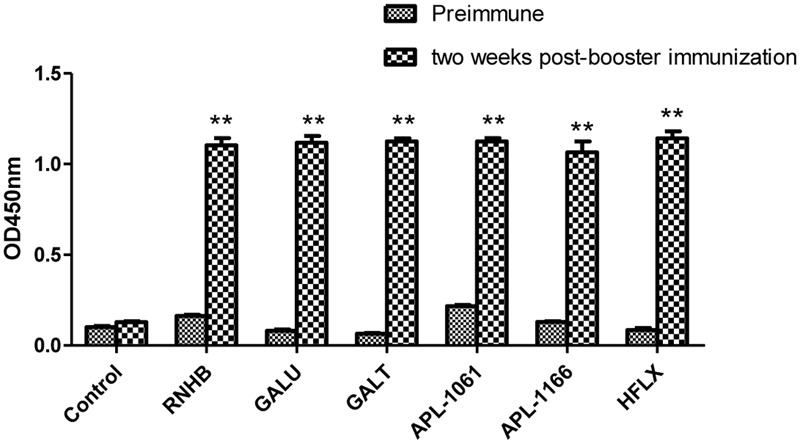
**Analysis of levels of IgG.** Serum samples from immunized controls and negative controls were collected before immunization and 2 weeks after booster immunization. Antibody levels were tested using an indirect ELISA coated with the corresponding recombinant proteins. Antibody levels were demonstrated as absorbance at 450 nm.

Two weeks after booster immunization, the serum samples of each group were collected and the IgG levels were tested by indirect ELISA. Referring to the results of indirect ELISA (**Figure 4**), IgG levels of every group were increased by relatively large margins compared with the serum before immunization. Compared with negative group, IgG levels of each IVI antigen protein group were significantly elevated (*P* < 0.001).

### Cell-Mediated Immune Response

**FIGURE 5:**
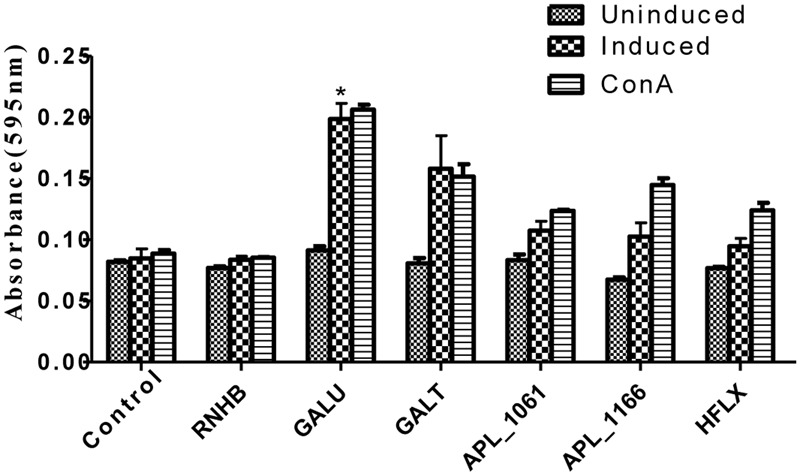
**Lymphocyte proliferation assay.** Levels of lymphocyte proliferation of each group were detected using an MTT method and the results shown as absorbance at 595 nm. Splenocytes of animals 2 weeks post-immunization were isolated and stimulated with recombinant proteins and con A.

Upon stimulation with recombinant proteins, except rRnhB group, levels of lymphocyte proliferation of all the proteins in the immune groups were higher than those of the negative control (**Figure 5**). Similarly, lymphocyte proliferation of these groups was also detected after the cells stimulated with ConA.

**FIGURE 6:**
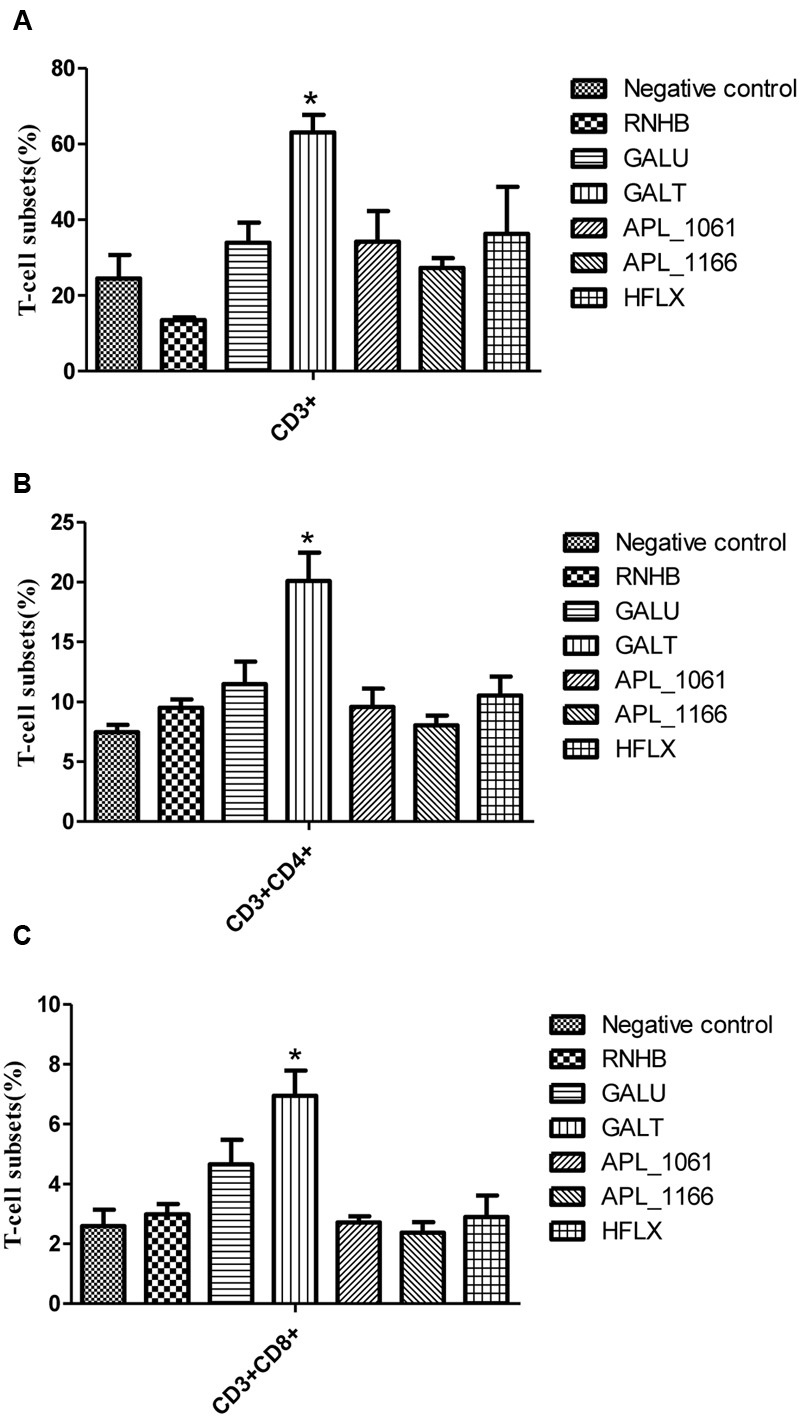
**FACS analysis.** The levels of CD3+ **(A)**, CD3+CD4+ **(B)**, and CD3+CD8+ **(C)** T-cell subsets in splenocytes from immunized groups and the negative control group were analyzed by FACS. The average measured percentages of CD3+, CD3+CD4+, and CD3+CD8+ T-cells with representative scatter diagrams of the T-cells subsets detected by flow cytometry method (data not shown). The means with standard deviations were represented as bars graph, and are representatives of the percentages of CD3+, CD3+CD4+, and CD3+CD8+ T-cells of spleens.

Distribution of CD3+, CD4+, and CD8+ T cells in the spleen cells were evaluated by FACS. Overall trends in the proportion of CD3+ and CD4+ T cells were higher in immunized mice than the control group animals (**Figure 6**). In rGalT group, all of CD3+, CD4+, and CD8+ showed the highest levels, which was significantly higher than the control group (*P* < 0.05).

**FIGURE 7:**
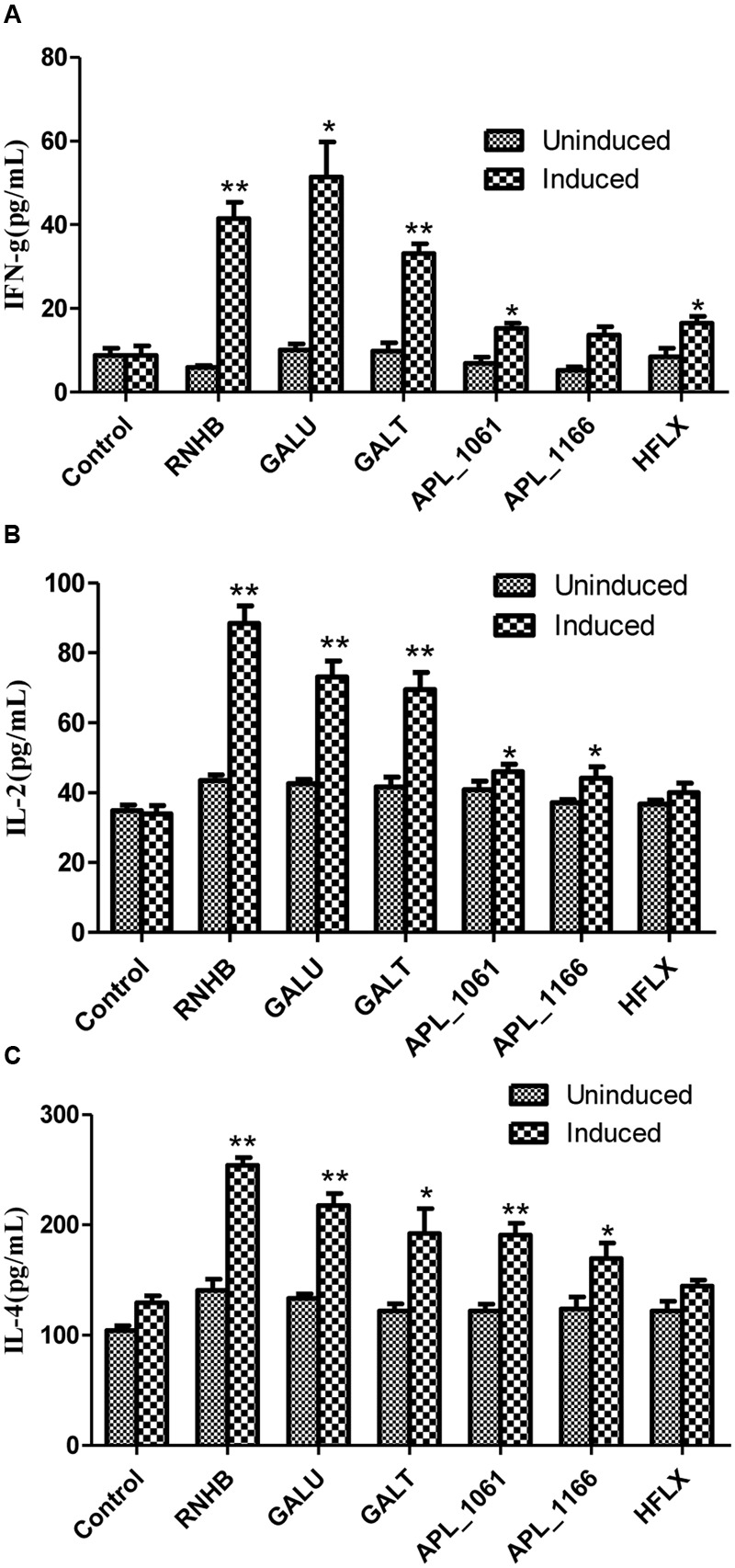
**The levels of IFN-γ **(A)**, IL-2 **(B)**, and IL-4 **(C)** in cultured spleen cells of mice injected with proteins or negative control were determined by sandwich ELISA**.

On the other hand, the levels of IFN-γ, IL-2 and IL-4 stimulated with recombinant proteins were significantly higher in the vaccinated groups when compared with the control group (*P* < 0.05; **Figure 7**). The levels of IL-4 in response to stimulation with recombinant proteins were higher than the level of IFN-γ and IL-2 (**Figure 7**). Except APL_1166 group, levels of IFN-γ of six immunized groups were significantly higher than the control (*P* < 0.05). With the exception of the HflX group, both levels of IL-2 and IL-4 of five immunized groups were significantly higher than the control group (*P* < 0.05).

### Protective Efficiency in Murine Model

**FIGURE 8:**
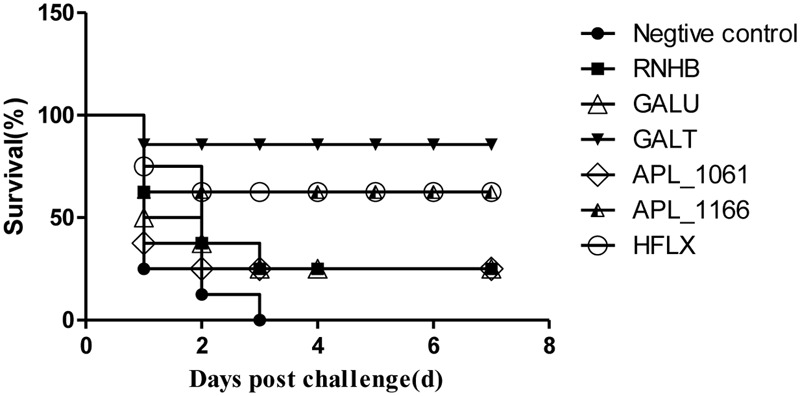
**Survival rate of mice challenged with APP strain L20 after immunization.** The animals immunized with recombinant vaccine were challenged after 2 weeks of last booster with 5 × 10^8^ CFU (10 LD50) of App L20, intraperitoneally. The animals were monitored for mortality till day 14 post-challenge. Over all there was significant difference in survival of control and vaccinated animals (*P* < 0.05). The figure shows the post-challenge survival as a representative vaccine evaluation and difference in time period between mortality of control and vaccine group.

The results of vaccination and challenge experiments showed that rGalT, rAPL_1166, and rHlfX provided 87.5, 62.5, and 62.5% protection, respectively (**Figure 8**). The mortality of mice in the negative control group was 100% after being challenged with APP L20. The rRnhB, rGalU, and rAPL_1061 immunized groups had an identical protection efficiency of 25% within the observation days (**Figure 8**). The survival rate of mice challenged with APP strain L20 after vaccination was shown in **Figure 8**.

### Histopathologic Analysis

**FIGURE 9:**
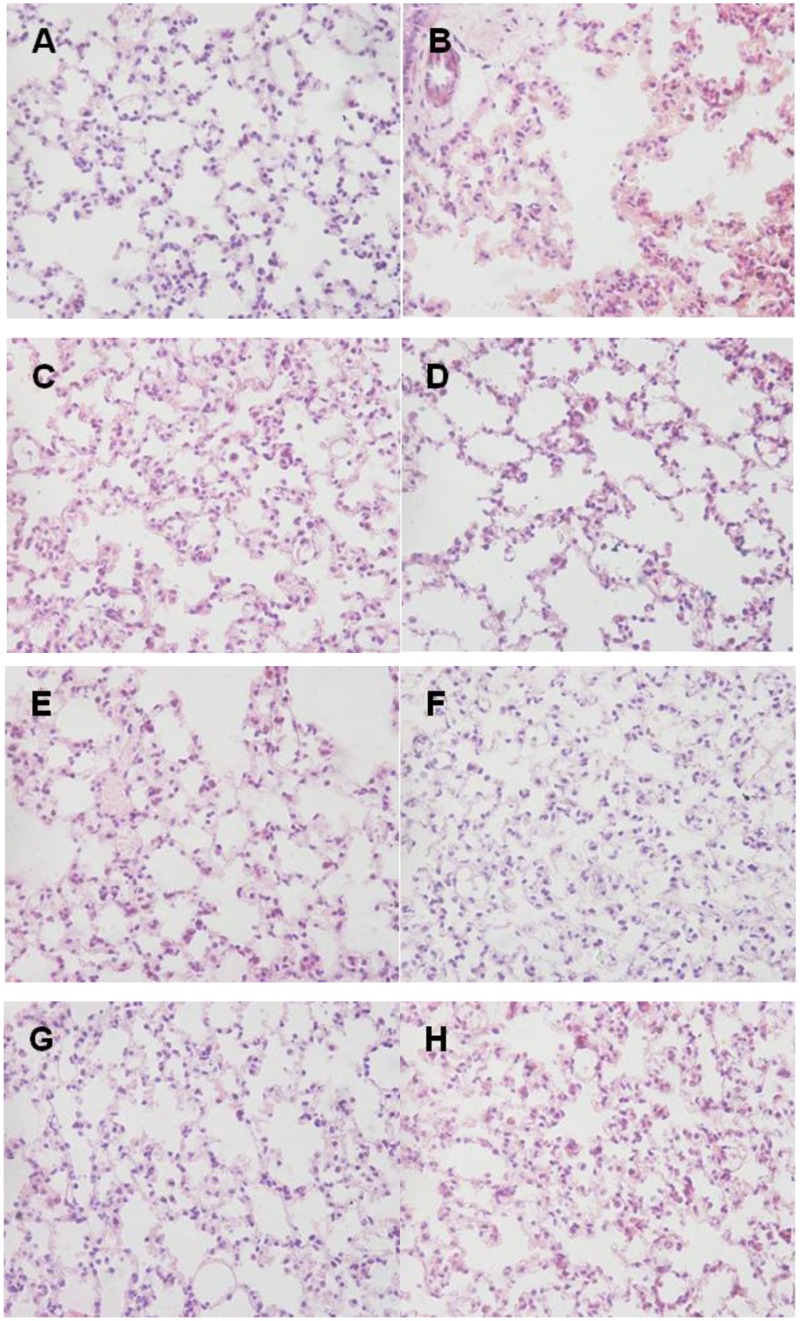
**Lung histopathology.** Mice of each group were sacrificed post-challenge and lungs were collected for histopathology. **(A)** Normal control. **(B)** Negative control. **(C–H)** Lungs from mice immunized with RnhB, GalU, GalT, APL_1061, APL_1166, and HflX, respectively. Hematoxylin and Eosin (H&E), magnification 400×. **(A)** Normal control. **(B)** Negative control mouse lung showing increased inflammatory cell infiltration in the perivascular and peribronchial areas. **(C)** Tissue from mice immunized with rRnhB showing a mild inflammatory cell infiltration in the perivascular and peribronchial areas. **(D)** Lung tissue from surviving infected mouse showing significantly reduced infiltration of inflammatory cells.

Histopathologic examination showed the infiltration of neutrophils and macrophages in the lung tissues (**Figure 9**). Lung tissue of mice in control group was severely damaged and the structures of pulmonary alveoli showed pathological changes (**Figure 9B**). Moreover, the lung parenchyma was edematous (**Figure 9B**). However, all surviving mice from the immunized groups did not manifest significant pathological damage (**Figure 9**). Immunized groups had only moderate inflammation with infiltration of mixed mononuclear cells and neutrophils (**Figures 9C–H**).

### Immunohistochemical Analysis

**FIGURE 10:**
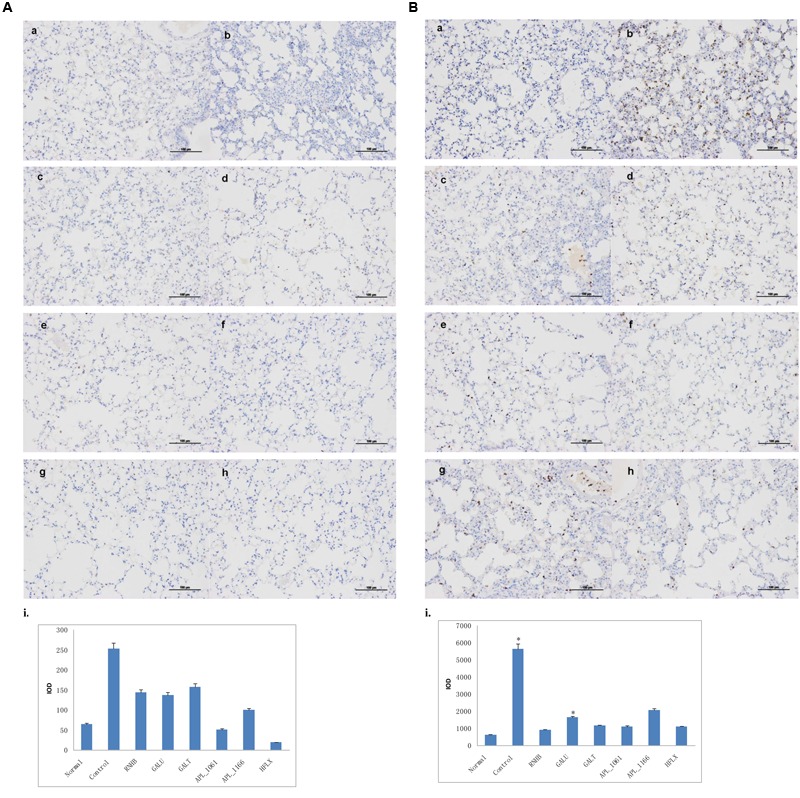
**Immunohistochemical analysis.** Mice of each group were sacrificed post-challenge and lungs were collected for histopathology. **(A)** Immunohistochemical **(IHC)** analysis for macrophages. **(B)** IHC analysis for neutrophils. **(a)** Normal control. **(b)** Negative control. **(c–h)** Lungs from mice immunized with RnhB, GalU, GalT, APL_1061, APL_1166, and HflX, respectively. **(i)** Statistical analysis of inflammatory cells.

Compared with IHC analysis results of normal control, IOD of neutrophils and macrophages in negative control group were significant higher than normal group (*P* < 0.05; **Figures 10A,B**). IOD of neutrophils in all immunized groups except GalU were not significantly different to that of the normal group. Nevertheless, neither IOD of negative control nor all immunized groups were significantly different with normal control (**Figure 10B**).

## Discussion

*Actinobacillus pleuropneumoniae* is one of the most important bacterial pathogens, which causes PCP (Li et al., 2016). For strategies aimed at controlling this disease, vaccines appear to be the most effective choice. Over the last several years, numerous studies have been performed with the goal of generating APP vaccines with improved protective efficacy rates. Inactivated whole-cell vaccines have been the standard means used to control PCP. These “traditional” vaccines have reduced, to some extent, mortality rates, but have shown little use in curbing morbidity rates. There was no cross-protection for hosts immunized with inactivated vaccine (Jolie et al., 1995). However, attenuated mutant vaccine candidates have been shown to provide a partial cross-protection and reduced morbidity (Tonpitak et al., 2002; Maas et al., 2006). So far, DNA vaccines (Chiang et al., 2009; Lu et al., 2011), subunit vaccines (Sjolund and Wallgren, 2010; Lopez-Bermudez et al., 2014), recombinant subunit vaccines (Jirawattanapong et al., 2008; Oldfield et al., 2008, 2009; Shao et al., 2010; Sadilkova et al., 2012; Seo et al., 2013; Park et al., 2015; Li et al., 2016), ghost vaccine (Katinger et al., 1999; Hensel et al., 2000), and live vector vaccine (Shin et al., 2013; Hur and Lee, 2014) for APP had been studied.

The current APP vaccines derive primarily from *apx*, omp and other key antigens, all of which are known virulence factors of APP; these vaccines so far have shown to provide partial protection. However, the current battery of APP vaccines does not protect against all APP pathogenic serovars. We have previously applied IVIAT to screen the genome of APP L20 and a number of IVI antigens were identified (Zhang et al., 2015). Compared with *in vitro* conditions, the levels of transcription of these antigens *in vivo* were up-regulated. Furthermore, these antigens were demonstrated to be conserved in different serovars. We hypothesized that the IVI antigens identified in our previous study were highly antigenic and could provide immune protection against APP. Among these antigens, APL_1061 and APL_1166 are APP antigens of unknown function. RnhB (Itaya, 1990) and HflX (John et al., 2005) are ribonuclease H and GTP-binding protein, respectively. GalU, UTP-α-D-glucose-1-phosphate uridylyltransferase is involved in the LPS core biosynthesis (Rioux et al., 1999), and GalU and GalT are enzymes involved in metabolism of galactose (Ebrecht et al., 2015), all essential for the virulence of different bacterial pathogens (Chai et al., 2012; Caboni et al., 2015).

We performed the vaccine trial using these antigens. Our studies indicated that IVI antigens conferred a partial protection in a mouse challenge model. Vaccinated groups showed high level antibody responses. Also, increased level of cytokines was detected in our vaccinated groups. Among these antigens, GalT, APL_1166, and HflX immunized groups showed relatively high levels of immune protection and could be considered as potential vaccine candidates for further study in piglets.

This is the first report to determine whether the IVI antigens could induce an immune response against App challenge in a mouse model. Both humoral and cellular immune responses were induced in the immunized animals using these IVI antigens as vaccine candidates. However, our recombinant IVI proteins could elicit a strong Th2 response. In conclusion, our results showed that IVI proteins can be used as protective antigens against APP in mice and should be considered as promising vaccine candidates for further studies in a piglet model.

## Author Contributions

FZ, SC, QZ, and XW conceived and designed the experiments. FZ, ZZ, YY, YW, and RW performed the experiments. FZ, SC, Y-FC, XH, YH, and XM analyzed the data. FZ, SC, QY, and Y-FC wrote the paper.

## Conflict of Interest Statement

The authors declare that the research was conducted in the absence of any commercial or financial relationships that could be construed as a potential conflict of interest.

## Footnotes

1 http://www.cbs.dtu.dk/services/SignalP/
